# Práticas seguras no manejo de vias aéreas de pacientes com Covid-19: revisão integrativa

**DOI:** 10.15649/cuidarte.1356

**Published:** 2021-08-20

**Authors:** Cristina da Silva-Fernandes, Maria Girlane Sousa Albuquerque-Brandão, Magda Milleyde de Sousa-Lima, Jennara Cândido-do-Nascimento, Nelson Miguel Galindo-Neto, Lívia Moreira-Barros

**Affiliations:** 1 Universidade Estadual Vale do Acaraú (UVA), Sobral-CE, Brasil, E-mail: cristina.sednanref@gmail. com Autor correspondente Universidade Estadual do Vale do Acaraú Universidade Estadual Vale do Acaraú (UVA) Brazil cristina.sednanref@gmail. com; 2 Universidade da Integração Internacional da Lusofonia Afro- Brasileira (UNILAB), Redenção-CE, Brasil. E-mail: girlane.albuquerque@yahoo. com.br Universidade da Integração Internacional da Lusofonia Afro- Brasileira (UNILAB) Brasil girlane.albuquerque@yahoo. com.br; 3 Universidade Federal do Ceará (UFC), Fortaleza-CE, Brasil. E-mail: limamilleyde@gmail.com Universidade Federal do Ceará Universidade Federal do Ceará (UFC) Brazil limamilleyde@gmail.com; 4 Universidade Federal do Ceará (UFC), Fortaleza-CE, Brasil. E-mail: jennaracandido@yahoo.com.br Universidade Federal do Ceará Universidade Federal do Ceará (UFC) Brazil jennaracandido@yahoo.com.br; 5 Instituto Federal de Educação, Ciência e Tecnologia de Pernambuco (IFPE), Pesqueira-PE, Brasil. E-mail: nelsonmiguelnt@hotmail.com Instituto Federal de Educação Brasil nelsonmiguelnt@hotmail.com; 6 Universidade Federal do Ceará (UFC), Fortaleza-CE, Brasil. E-mail: livia.moreirab@hotmail.com Universidade Federal do Ceará Universidade Federal do Ceará Brazil livia.moreirab@hotmail.com

**Keywords:** Enfermagem, Betacoronavirus, Manuseio das Vias Aéreas, Saúde do Trabalhador., Nursing, Betacoronavirus, Airway Management, Occupational Health, Enfermería, Betacoronavirus, Manejo de la Vía Aérea, Salud Laboral

## Abstract

**Introdução:**

A pandemia causada pelo vírus SARS-CoV-2 no início de 2020 alterou práticas dos profissionais de saúde com a finalidade em atenuar os riscos de contaminação dos trabalhadores da linha de frente de assistência à saúde, principalmente, atividades relacionadas ao manejo de vias aéreas de pacientes com Covid-19.

**Objetivo:**

identificar as práticas necessárias para garantir a segurança dos profissionais de saúde no manejo de vias aéreas de pacientes suspeitos ou diagnosticados com Covid-19.

**Materiais e métodos:**

revisão integrativa realizada em dez bases de dados relevantes na área da saúde, sendo a amostra composta por 17 pesquisas. As práticas identificadas foram elencadas conforme os quatro procedimentos pontuados pela literatura: intubação endotraqueal, extubação, broncoscopia e traqueostomia.

**Resultados:**

as principais recomendações mencionadas foram: intubação endotraqueal: realizada por equipe experiente, treinada, mínima e profissionais de grupos de risco para Covid-19 não devem fazer parte desta equipe; extubação: não utilizar cateter nasal de alto fluxo após o procedimento; broncoscopia: realizar em sala isolada e com pressão negativa; traqueostomia: poderá ser considerada precocemente, mas o risco-benefício deve ser avaliado.

**Conclusão:**

as práticas identificadas poderão direcionar o gerenciamento de vias aéreas e nortear a construção de tecnologias assistenciais, educacionais ou gerenciais.

## Abstract (en)

**Introduction:**

The pandemic caused by the SARS-CoV-2 virus in early 2020 changed the practices of health professionals in order to mitigate the risks of contamination of health care frontline workers, mainly activities related to the management of the airways of patients with Covid-19.

**Objective:**

to identify the practices necessary to ensure the safety of health professionals in managing the airway of patients suspected or diagnosed with Covid-19.

**Materials and methods:**

an integrative review carried out in ten relevant databases in the health area, with the sample consisting of 17 surveys. The practices identified were listed according to the four procedures punctuated by the literature: endotracheal intubation, extubation, bronchoscopy and tracheostomy.

**Results:**

the main recommendations mentioned were: endotracheal intubation: performed by an experienced, trained, minimal team and professionals from risk groups for covid-19 should not be part of this team; extubation: do not use a high flow nasal catheter after the procedure; bronchoscopy: perform in an isolated room with negative pressure; tracheostomy: may be considered early, but the risk-benefit must be assessed.

**Conclusion:**

the practices identified may direct the management of airways and guide the construction of assistive, educational or managerial technologies.

## Resumen

**Introducción:**

La pandemia causada por el virus SARS-CoV-2 a principios de 2020 cambió las prácticas de los profesionales de la salud con el fin de mitigar los riesgos de contaminación de los trabajadores de primera línea de atención médica, principalmente actividades relacionadas con el manejo de las vías respiratorias de pacientes con Covid-19.

**Objetivo:**

identificar las prácticas necesarias para garantizar la seguridad de los profesionales de la salud en el manejo de la vía aérea de pacientes sospechosos o diagnosticados con Covid-19.

**Materiales y métodos:**

una revisión integradora realizada en diez bases de datos relevantes en el área de la salud, la muestra consiste en 17 encuestas. Las prácticas identificadas se enumeraron de acuerdo con los cuatro procedimientos puntuados por la literatura: intubación endotraqueal, extubación, broncoscopia y traqueotomía.

**Resultados:**

las principales recomendaciones mencionadas fueron: intubación endotraqueal: realizada por un equipo experimentado, capacitado, mínimo y profesionales de grupos de riesgo para Covid-19 no deberían formar parte de este equipo; extubación: no use un catéter nasal de alto flujo después del procedimiento; broncoscopia: realizar en una habitación aislada con presión negativa; traqueotomía: puede considerarse temprano, pero se debe evaluar el riesgo-beneficio.

**Conclusión:**

las prácticas identificadas pueden dirigir la gestión de las vías aéreas y guiar la construcción de tecnologías de asistencia, educación o gestión.

## Introdução

A Doença de Coronavírus surgida em 2019 (Covid-19), causada por novo coronavírus (SARS- CoV-2), é uma patologia altamente contagiosa, transmitida por gotículas, contato ou aerossóis, que se espalhou para vários países por meio de pessoas infectadas, causando proporções pandémicas(1). Os sintomas clínicos incluem febre, tosse, fadiga e raramente, infecção gastrointestinal. Idosos e pessoas com comorbidades são mais suscetíveis e vulneráveis ao desenvolvimento de complicações como a Síndrome do Desconforto Respiratório Agudo (SDRA)(2),(3).

A SDRA e pneumonia bilateral são os principais desafios no tratamento de pacientes diagnosticados com Covid-19. Muitos pacientes entrarão em estado crítico e haverá necessidade de intubação, para manter os parâmetros fisiológicos da função respiratória, o que exige contato próximo dos profissionais de saúde(2,4,5). Entretanto, a carga viral nas vias aéreas de pessoas com o novo coronavírus é, provavelmente, elevada e contagiosa(5),(6) , o que representa riscos significativos para aqueles que realizam o manejo de vias aéreas(7),(8).

Assim, os profissionais de saúde enfrentam elevado risco de exposição e infecção(3). Em 25 de fevereiro de 2020, a China relatou 3387 profissionais de saúde infectados apenas em Hubei, dos quais, pelo menos 18 vieram a óbito(9). Até 9 de abril de 2020, 9.282 (19%) casos de Covid -19 foram identificados em profissionais de saúde dos Estados Unidos(10).

Apesar do alto risco de contaminação, as condutas clínicas sobre o manejo das vias aéreas e recomendações de especialistas nesses pacientes ainda são escassas e urgentemente necessárias(8). Para garantir a segurança do paciente e dos profissionais envolvidos no manejo de vias aéreas são necessárias considerações e precauções especiais(7). Frente ao exposto e com a pandemia do novo coronavírus atualmente em curso, surgiu o questionamento: “Quais as práticas necessárias para garantir a segurança dos profissionais de saúde no manejo de vias aéreas de pacientes com Covid-19?”.

É imprescindível identificar quais são as práticas de cuidado durante ou manejo das vias aéreas dos pacientes como novos coronavírus, para que medidas preventivas e práticas seguras sejam previamente protocolizadas e implementadas, como forma de reduzir a transmissão do vírus e garantir a segurança dos profissionais de saúde durante o atendimento.

Nessa perspectiva, o presente estudo tem o objetivo de identificar as práticas necessárias para garantir a segurança dos profissionais de saúde e o manejo das vias aéreas de pacientes suspeitos ou diagnosticados com Covid-19.

## Materiais e métodos

O método de síntese utilizado foi a revisão integrativa, a qual reúne, avalia e sintetiza os resultados de pesquisas sobre temática específica, cuja realização ocorreu no período de abril a maio de 2020. Para condução dessa investigação, percorreram-se cinco etapas: elaboração da questão norteadora (identificação do problema), busca dos estudos na literatura, avaliação dos estudos, análise dos dados e apresentação da revisão(11).

A questão norteadora do estudo “Quais as práticas necessárias para garantir a segurança dos profissionais de saúde no manejo de vias aéreas de pacientes com Covid-19? ” foi construída com auxílio da estratégia PICO, sendo P de população, paciente ou problema (profissionais de saúde), I de intervenção (manejo de vias aéreas de pacientes com Covid-19) e o elemento O (desfecho) representado pela segurança laboral. Enfatiza-se que o elemento C, de comparação entre intervenção ou grupo, não foi empregado pelo tipo de revisão desenvolvida(12).

Para a busca dos estudos primários foram selecionadas as bases de dados Cinahl (Cumulative Index to Nursing and Allied Health Literature), Scopus, Web of Science, Science Direct, Embase, Medrxiv, acessadas pelo Portal Periódicos Capes; Medline (Medical Literature Analysis and Retrieval Sistem on-line) e PubMed Central, acessadas pela Pubmed; portal Scielo e biblioteca Cochrane.

Em cada base de dados, os descritores controlados foram delimitados pelo Descritores em Ciências da Saúde (DECS) e MeSH (Medical Subject Headings). Foi empregado o cruzamento: (“Covid 19”OR Covid-19 OR corona OR coronavírus OR sars-cov-2) AND (“Intratracheal Intubation” OR “Tracheal Intubation” OR “Airways” OR “airway aspiration”) AND (“clinical management” OR “Patient Safety” OR “Care safety”).

Os critérios de inclusão foram publicações divulgadas em 2020, sem delimitação de idioma, e que abordassem recomendações sobre o manejo de vias aéreas em pacientes diagnosticados ou com suspeita de Covid-19. Foram excluídas teses, dissertações, editoriais, publicações que não possuíam relação direta com o tema e duplicadas.

Na avaliação dos estudos, a nomenclatura relativa ao tipo de estudo indicada pelos autores foi mantida. Quando o tipo de estudo não foi descrito de forma clara pelos pesquisadores, a análise foi fundamentada nos conceitos sobre metodologia científica de pesquisadores da enfermagem(13). Assim, foram considerados para a amostra, estudos que apresentassem qualidade metodológica, evidências científicas fundamentadas e fossem atualizados quanto às referências e recomendações relacionadas ao objeto de estudo.

Segundo a questão clínica do estudo, pesquisadores propuseram hierarquias de evidências, que foram adotadas na presente revisão para classificar a força de evidência. Dessa forma, a questão clínica do estudo pode ser de Intervenção/Tratamento ou Diagnóstico/Teste diagnóstico(14).

A força da evidência pode ser classificada em sete níveis, nos quais o mais forte (nível 1) às evidências de revisão sistemática ou metanálise de todos os ensaios clínicos randomizados relevantes. Quando a questão clínica de Prognóstico/Predição ou Etiologia, a força da evidência pode ser classificada em cinco níveis, nos quais o mais forte (nível I) consiste nas evidências de síntese de estudos de coorte ou de caso-controle. Com relação à questão clínica sobre Significado, a força da evidência pode ser classificada em cinco níveis, sendo o mais forte (nível I) as evidências de metassíntese de estudos qualitativos(14).

A extração dos dados dos estudos primários foi executada com o subsídio de instrumento elaborado e submetido à validação aparente e de conteúdo(15). A análise dos dados da revisão integrativa foi elaborada na forma descritiva. Para cada estudo incluído, elaborou-se quadro- síntese composto pelas seguintes variáveis: título do artigo, autor (es), periódico, ano de publicação, tipo de estudo, principais resultados e conclusões, o qual permitiu a comparação das diferenças e similaridades entre as pesquisas e organização dos dados.

Os aspectos éticos e legais foram respeitados. Os estudos incluídos na pesquisa tiveram os nomes dos seus autores devidamente referenciados.

## Resultados

A partir da busca nas bases de dados, foram encontradas 389 publicações: PubMed Central= 34 Medline=2; Science Direct=339; Scopus=19; Cinahl=0; Web of Science=1; Embase=1; Medrxiv=0 Cochrane=0; Scielo=0.

**Figura 1:**
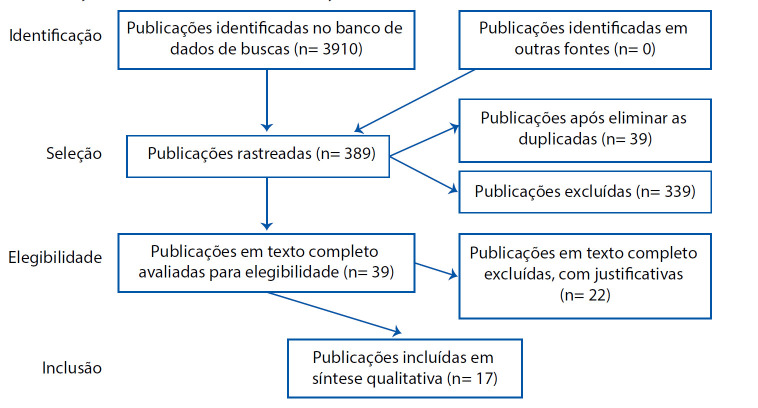
Fluxograma do processo de seleção dos estudos adaptado do Preferred Reporting Items for Systematic Review and Meta-Analyses (PRISMA)

Após a leitura do título e resumo de cada publicação, 11 eram duplicadas e foram excluídas. Os demais artigos (n=378), após aplicação dos critérios de seleção, foram excluídas 339 publicações que não se tinham relação com o objeto da pesquisa. Em seguida 39 publicações foram classificadas como potencialmente elegíveis, às quais foram analisadas na íntegra. Em seguida, 22 estudos foram excluídos, pois alguns não respondiam a questão norteadora e outros não apresentavam de forma explícita o delineamento da pesquisa. Assim, 17 estudos compuseram a amostra da revisão, conforme Figura 1.

Fonte: elaboração pelos autores (2020)

Dos 17 estudos selecionados, onze foram classificados com tipo de questão clínica Intervenção/Tratamento ou Diagnóstico/Teste diagnóstico, sendo dois com nível de evidência III, três com nível VI e seis com nível VII; seis foram classificados com tipo de questão clínica Prognóstico/Predição ou Etiologia, sendo um com nível de evidência III, dois com nível de evidência VI e três com nível V. A caracterização dos estudos apresenta-se no Quadro 1.

**Quadro 1:** Caracterização dos estudos, segundo autor (es), ano de publicação, tipo de estudo, questão clínica e nível de evidência, (n=17), Sobral, Ceará, Brasil, 2020

Autores	Ano	Tipo de estudo	Questão clínica - nível de evidência
Kluge *et al*(16)	2020	Diretrizes de especialistas	Prognóstico/Predição - V*
Xu *et al*(17)	2020	Diretrizes de especialistas	Prognóstico/Predição - V*
Sorbello *et al*(18)	2020	Diretrizes de especialistas	Intervenção/Tratamento - VII†
Luo *et al*(7)	2020	Diretrizes de especialistas	Intervenção/Tratamento - VII†
Yao *et al*(8)	2020	Diretrizes de especialistas	Prognóstico/Predição - IV§
Chávez *et al*(19)	2020	Revisão narrativa	Intervenção/Tratamento - VI*
Sanz *et al*(20)	2020	Diretrizes de especialistas	Prognóstico/Predição - V*
Nicola *et al*(21)	2020	Revisão narrativa	Prognóstico/Predição - IV§
Pichi *et al*(22)	2020	Diretrizes de especialistas	Intervenção/Tratamento - VII†
Phua *et al*(23)	2020	Revisão narrativa	Prognóstico/Predição - III§
Lavinsky *et al*(24)	2020	Revisão narrativa	Intervenção/Tratamento - III†
Lyons *et al*(25)	2020	Revisão narrativa	Intervenção/Tratamento - III†
Senturk *et al*(26)	2020	Diretrizes de especialistas	Intervenção/Tratamento - VII†
Pérez *et al*(27)	2020	Diretrizes de especialistas	Intervenção/Tratamento - VII†
Huang *et al*(28)	2020	Diretrizes de especialistas	Intervenção/Tratamento - VII†
Mei *et al*(29)	2020	Revisão narrativa	Diagnóstico/Teste diagnóstico - VI*
D’Silva *et al*(30)	2020	Revisão narrativa	Intervenção/Tratamento - VI*

*V- Evidência proveniente de opinião de especialistas; †VII- Evidências oriundas de opinião de autoridades e/ou relatório de comitês de especialistas; §IV- Evidências de um único estudo qualitativo ou descritivo; *VI- Evidências derivadas de um único estudo descritivo ou qualitativo; †III - Evidências de ensaios clínicos bem delineados sem randomização; §III- Evidência de metassíntese de estudos qualitativos ou descritivos.

Fonte: elaboração pelos autores (2020)

A partir da leitura completa dos estudos que compuseram a amostra da revisão, foram elencadas as recomendações de práticas para segurança dos profissionais de saúde, no manejo de vias aéreas de pacientes com Covid-19. As sugestões de condutas foram enumeradas conforme os procedimentos descritos na literatura científica e encontram-se apresentadas nos Quadros 2, 3, 4 e 5.

O procedimento mais comum no manejo de vias aéreas foi a intubação endotraqueal. As principais recomendações descritas durante esse procedimento são: ser realizada por equipe experiente, treinada, mínima e profissionais dos grupos de risco para Covid-19 não devem fazer parte da equipe (Quadro 2).

**Quadro 2:** Recomendações para práticas de garantia da segurança dos profissionais de saúde na intubação endotraqueal de pacientes com Covid-19. Sobral, Ceará, Brasil, 2020.

Recomendações na Intubação Endotraqueal Artigo (n=15)	Recomendações na Intubação Endotraqueal Artigo (n=15)
Deve ser realizada por equipe experiente, treinada e mínima. Ademais, profissionais de grupos de risco para Covid-19 não devem fazer parte da equipe.	Kluge et al., 2020(16); Sorbello et al., 2020(18); Yao et al., 20208; Chávez et al., 2020(19); Nicola et al., 2020(21); Phua et al., 2020(23); Senturk et al., 2020(26)
Deve ser realizada em sequência rápida sem ventilação intermediária.	Kluge et al., 2020(16); Xu et al., 2020(17); Sorbello et al, 2020(18); Luo et al., 20207; Yao et al., 20208; Chávez et al., 2020(19); Sanz et al., 2020(20)
Utilizar videolaringoscópio com tela remota separada, se disponível.	Kluge et al16., 2020; Sorbello et al., 2020(18); Luo et al., 20207; Yao et al., 20208; Lyons et al., 2020(25); Senturk et al., 2020(26)
Não realizar ausculta para verificação de localização correta do tubo.	Kluge et al., 2020(16); Sorbello et al., 2020(18); Luo et al., 20207
Se necessitar de Ressuscitação Cardiopulmonar (RCP), a equipe deverá ser mínima e treinada.	Kluge et al., 2020(16)
Devem ser utilizados relaxantes musculares adequados e com ação e eficácia rápidas.	Xu et al., 2020(17); Sorbello et al., 2020(18); Phua et al., 2020(23); Senturk et al., 2020(26)
Adoção de precauções grau III: máscaras (N95 ou N99); roupa de proteção; roupa de trabalho; isolamento anti-pe-netração; bata; três luvas de látex; capas para sapatos; protetores anti-embaciamento; óculos ou escudo prote-tor; ampla máscara facial ou respiradores purificadores de ar elétricos (PAPR), se disponível	Xu et al.17, 2020; Luo et al., 20207; Yao et al., 20208; Chávez et al., 2020(19); Senturk et al., 2020(26); Huang et al., 2020(28)
O procedimento precoce é incentivado, no entanto o risco-benefício deverá ser criteriosamente avaliado.	Sorbello et al., 2020(18); Lavinsky et al., 2020(24); Senturk et al., 20202
Utilizar listas de verificação antes, durante e após o proce-dimento.	Sorbello et al., 2020(18)
Realizar intubação em sala com pressão negativa, se dispo-nível.	Sorbello et al., 2020(18); Senturk et al., 2020(26); Huang et al., 2020(28)
A ventilação com Bolsa-Válvula-Máscara (BVM) deve ser utilizada durante o procedimento, se extremamente necessário em casos de dessaturação grave.	Sorbello et al., 2020(18); Chávez et al., 2020(19); Sanz et al., 2020(20); Phua et al., 2020(23)
Cânula nasal de alto fluxo não deve ser utilizada para pré-oxigenação.	Sorbello et al., 2020(18)
Dispositivos supraglóticos de segunda geração são recomendados em caso de duas tentativas falhas.	Sorbello et al., 2020(18); Yao et al., 20208
O tempo do procedimento deve ser menor que 20 segun-dos.	Luo et al., 2020(7)
Máscara laríngea deve ser inserida após uma falha.	Luo et al., 2020(7)
Uma equipe multidisciplinar extra deve ficar disponível e paramentada fora da sala de intubação.	Luo et al., 2020(7); Senturk et al., 2020(26)
Não realizar classi_cação de Mallampati	Yao et al., 2020(8)
A colocação e retirada dos EPIs devem ser observadas por outro profissional.	Yao et al., 2020(8); Senturk et al., 2020(26); Huang et al., 2020(28)
Intubações no pré-hospitalar devem ser evitadas.	Chávez et al., 2020(19)
Em intubações difíceis, recomenda-se cricotireoidotomia.	Lavinsky et al., 2020(24)
A pré-oxigenação deve ser realizada com máscara bem ajustada e com Mapleson C ('Waters').	Senturk et al., 2020(26)
A aspiração pós-intubação deve ser realizada em sistema fechado com ponta de cateter infraglótico.	Senturk et al., 2020(26)
Estabelecer rodízio entre as equipes de intubação.	Huang et al., 2020(28)

Fonte: elaboração pelos autores (2020)

Durante a extubação do paciente, assim como na intubação, deve ser empregado o mesmo rigor de precaução, nível de EPI’s, condições e logística, dentre outras recomendações de segurança, detalhadas no Quadro 3.

**Quadro 3:** Recomendações para práticas de garantia da segurança dos profissionais de saúde durante extubação de pacientes com Covid-19. Sobral, Ceará, Brasil, 2020.

Recomendações na Extubação	Artigo (n=2)
Antes da extubação, deve-se realizar aspiração por sistema fechado e cateter infraglótico.	Senturk et al., 2020(26)
Não utilizar cateter nasal de alto fluxo após extubação.	Senturk et al., 2020(26); D’Silva et al., 2020(30)
Utilizar mesmo grau de precaução da intubação.	Senturk et al., 2020(26)
Utilizar medicamentos e_cazes na diminuição da tosse.	Senturk et al., 2020(26); D’Silva et al., 2020(30)
Colocar máscara cirúrgica ou N95 no paciente após extubação. Esperar 20 minutos para desinfectar o local após extubação.	Senturk et al., 2020(26)
Deve ocorrer em sala com pressão negativa.	D’Silva et al., 2020(30)
A equipe presente na sala deve ser mínima.	
A entrada de pessoal na sala deve ocorrer 30 minutos após o procedimento.	
Utilizar técnica “Mask Over Tube”.	
Não aplicar nenhuma oxigenação com pressão positiva durante extubação.	
Extubar na expiração final.	

Fonte: elaboração pelos autores (2020)

Durante procedimento de broncoscopia nos pacientes com Covid-19 devem ser seguidas rigorosamente as medidas básicas de controle de infecção, diante do risco de exposição a aerossóis (Quadro 4).

**Quadro 4:** Recomendações para práticas de garantia da segurança dos profissionais de saúde durante broncoscopia de pacientes com Covid-19. Sobral, Ceará, Brasil, 2020.

Recomendações na Broncoscopia	Artigo (n=4)
Realizar, se absolutamente necessário.	Kluge et al., 2020(16)
Realizar broncoscopia com _bra óptica.	Lyons et al., 2020(25)
Deve ser realizada com EPI adequado, incluindo luvas, casacos impermeáveis de mangas compridas, máscara FFP3 e proteção ocular adequada para o rosto.	Pérez et al., 2020(27)
Utilizar sedação a dequada.	
Paciente deve utilizar máscara cirúrgica sobre sistemas de oxigenoterapia.	
Em broncoscopia básica, a equipe deve ser mínima: endoscopista, enfermeiro e técnico assistente deenfermagem (TCAE). Na broncoscopia avançada: além do exposto, um anestesista. Na broncoscopiade UTI: além do exposto, um intensivista. A equipe deve ser experiente e treinada.	
Deve ser realizada em sala isolada e com pressão negativa.	Pérez et al., 2020(27); Mei et al., 2020(29)
Realizar aspiração com pressão negativa. Utilizar broncoscópios descartáveis.	Pérez et al., 2020(27)

Fonte: elaboração pelos autores (2020)

No Quadro 5, são descritas recomendações de segurança durante o procedimento de traqueostomia.

**Quadro 5:** Recomendações para práticas de garantia da segurança dos profissionais de saúde durante traqueostomia de pacientes com Covid-19. Sobral, Ceará, Brasil, 2020.

Recomendações na Traqueostomia	Artigo (n=3)
Poderá ser considerada precocemente, mas o risco-benefício deve ser avaliado.	Luo et al., 2020(7); Lavinsky et al., 2020(24)
Necessário utilizar óculos ou escudo facial; vestido duplo, quando disponível; luvas duplas de nitrilo; cobertura adicional, como capuzes cirúrgicos. Utilizar máscaras FFP3 ou N99, no caso de falta de máscara FFP3; as máscaras FFP2 ou N95 podem ser utilizadas cobertas com máscara cirúrgica.	Pichi et al., 2020(22)
Na sala de procedimento deverá ficar apenas médicos e enfermeiros.	
Utilizar bloqueadores neuromuscular e_cazes e de rápida ação.	
O manuseio da cânula traqueal deve ser realizado em ambiente BSL-3.	
A troca do curativo deve ser realizada apenas em casos de vazamento do estoma.	
Em casos de cricotireoidotomia, considerar realização de traqueostomia após estabilização de vias aéreas.	Lavinsky et al., 2020(24)
Evitar usar aparelhos elétricos ou ultrassônicos.	
Realizar em sala com pressão negativa, se possível.	

Fonte: elaboração pelos autores (2020)

## Discussão

A Covid-19 é uma doença que causa deficiência nas estruturas do aparelho respiratório, o que leva a deficiências de funções da respiração e necessidade de manejo das vias aéreas para maximizar as taxas de sobrevida dos pacientes internados(31). Contudo, o manejo das vias aéreas de pacientes com Covid‐19 é um procedimento de alto risco, mediante a probabilidade de formação de aerossóis, o que aumenta potencialmente o risco de infecção entre os profissionais de saúde(32)^.^

Neste estudo, foram apresentadas recomendações que podem contribuir com a redução de transmissão do vírus e contribuir com a segurança dos profissionais de saúde durante manejo de vias aéreas avançadas, como intubação e extubação endotraqueal, broncoscopia e traqueostomia.

O procedimento mais comum no manejo de vias aéreas nessa clientela foi a intubação endotraqueal. Isso pode estar relacionado ao fato de o vírus SARS-COV-2 promover déficit nos parâmetros fisiológicos da função respiratória dos pacientes e culminar na necessidade de ventilação mecânica.

Pesquisa na China indicou que 14% dos pacientes com Covid-19 desenvolveram dispneia, taquipneia, dessaturação periférica de oxigênio (Spo2) menor ou igual a 93%, índice de oxigenação deficiente com uma razão Pao2/Fio2 <300 mmHg em 48hrs(33). Tais complicações podem levar a insuficiência respiratória hipoxêmica aguda que requer terapias de oxigênio e ventilação mecánica(34), (35).

As principais recomendações sobre intubação para ventilação artificial descritas nos estudos foram realização do procedimento por equipe experiente, treinada, mínima e exclusão de profissionais dos grupos de risco para Covid-19(8),(16),(18),(19),(21),(23),(26). Esses achados justificam-se uma vez que profissionais mais experientes e/ou treinados tendem a realizar menos de tentativas para sucesso na intubação orotraqueal. Além disso, a menor quantidade de profissionais presentes para o procedimento, resulta em menor exposição da equipe, aos riscos biológicos dos aerossóis.

Ademais, deve ser realizada em sequência rápida sem ventilação intermediária, pois o uso de ventilação com pressão positiva durante o procedimento aumenta as chances de aerossolização(7),(8),(16)^-^(20). Tais aerossóis, por permanecerem mais tempo suspensos no ar, constituem maior risco ocupacional para a equipe que realizou assistência ao paciente e para as pessoas que utilizarão o mesmo espaço físico, posteriormente.

Assim como na intubação, o processo de extubação do paciente com Covid-19 carece de precauções especiais, pois, para os profissionais de saúde intensivistas, é um dos momentos que representa o maior risco de exposição, por envolver contato direto com gotículas respiratorias(36), (37).

Os pacientes que necessitam de intubação para insuficiência respiratória ou cirurgia de emergência provavelmente permanecerão infecciosos no momento da extubação(38). Assim, é necessário rigor de precaução com uso adequado de EPI’s, que são essenciais para redução do risco de transmissão viral aos prestadores de serviços de saúde(30). Assim, é necessário esforço coordenado, com total apoio dos gestores hospitalares, para garantir os insumos necessários para manejo de vias aéreas(39).

Durante a broncoscopia, também pode haver a formação de aerossóis e esse procedimento só deve ser realizado se absolutamente necessário, seguindo recomendações específicas(16),(29) (Kluge et al., 2020; Pérez et al., 2020; Mei et al., 2020). As medidas de proteção contra aerossóis infecciosos derivados da broncoscopia são: uso de proteção respiratória no nível do respirador, salas de pressão negativa sempre que possível, além de equipe mínima e treinada(27),(29),(40).

Pesquisa nos Estados Unidos, que sumarizou diretrizes de sociedades de pneumologia em relação à broncoscopia, infere que todas as diretrizes sugerem que a broncoscopia é relativamente contraindicada na Covid‐19 e desempenha papel limitado no diagnóstico e tratamento da doença(29),(40).

Outro procedimento incluído no manejo de vias aéreas é a traqueostomia. Pesquisadores italianos revelaram que, com o cenário de pandemia do novo coronavírus, está ocorrendo aumento esperado dos procedimentos de traqueostomia(22). Pacientes em Unidades de Terapia Intensiva (UTI) sedados, que necessitam de intubação prolongada, geralmente requerem manejo mais seguro e prolongado das vias aéreas, que constitui indicação para a traqueostomia(29),(40).

Revisão sistemática realizada por pesquisadores do Egito revelou que a traqueostomia precoce, realizada nos primeiros sete dias após a intubação orotraqueal, está associada a redução da duração da ventilação mecânica, taxa de mortalidade e tempo de permanência na UTI(41). Contudo, é válido inferir que, devido ao acesso direto às vias aéreas, a traqueostomia pode gerar elevada quantidade de gotículas e apresenta maior probabilidade de formação de secreções, o que demanda manipulação frequente, para troca constante de curativo.

Dessa forma, o procedimento deve ser realizado mediante avaliação do risco-benefício, com utilização correta de EPI’s, equipe pequena e restrita, em sala com pressão negativa, se possível, com uso de bloqueadores neuromuscular eficazes e de rápida ação, com observação da necessidade de troca do curativo apenas em casos de vazamento do estoma(7),(22),(24).

Devido à formação de aerossóis, os procedimentos nas vias aéreas (intubação, extubação, broncoscopia, traqueotomia, entre outros) só devem ser realizados com medidas de proteção adequadas (incluindo máscara FFP2/FFP3 e óculos de proteção) e se for absolutamente necessário(16). Todas as medidas proteção-padrão devem, portanto, ser tomadas para minimizar os riscos à prestação de serviços de saúde, particularmente para manter o número de profissionais de saúde capazes de gerenciar a significativa população esperada de pacientes críticos(18).

As limitações do presente estudo relacionam-se, principalmente, ao delineamento das publicações incluídas na amostra da revisão, tendo em vista que a maioria trata-se de pesquisas descritivas, com a ausência de métodos experimentais. Ademais, alguns procedimentos, como a aspiração de vias aéreas, e oxigenoterapia, apesar de ser comum na prática de pacientes críticos com Covid-19, não foram identificadas recomendações que norteiam sua prática.

## Conclusão

As práticas necessárias para garantir a segurança dos profissionais de saúde no manejo de vias aéreas de pacientes com Covid-19 foram elencadas conforme os principais procedimentos identificados, os quais destacam-se a intubação endotraqueal, extubação, broncoscopia e traqueostomia.

Assim, pode-se considerar que a intubação endotraqueal deve ser realizada por equipe experiente, treinada, mínima e que profissionais de grupos de risco para Covid-19 não devem fazer parte desta equipe; na extubação não é recomendado utilizar cateter nasal de alto fluxo após o procedimento; a broncoscopia deve ser realizada em sala isolada e com pressão negativa; a traqueostomia poderá ser considerada precocemente, mas o risco-benefício deve ser avaliado.

As recomendações verificadas neste estudo poderão subsidiar o gerenciamento de vias aéreas, bem como, direcionar o desenvolvimento de tecnologias assistenciais, educacionais ou gerenciais que auxiliem o processo de cuidar e fomentem a cultura de segurança, com ênfase na atenuação do risco para o profissional de saúde e redução de iatrogenias.
